# Relationship between Lower Limb Kinematics and Upper Trunk Acceleration in Recreational Runners

**DOI:** 10.1155/2020/8973010

**Published:** 2020-01-16

**Authors:** Laura Simoni, Silvia Pancani, Federica Vannetti, Claudio Macchi, Guido Pasquini

**Affiliations:** Don Carlo Gnocchi Foundation IRCSS, 269 Via di Scandicci, 50143 Florence, Italy

## Abstract

Upper trunk (UT) kinematics in runners and its relationship with lower limbs has been poorly investigated, although it is acknowledged that dynamic stability of the upper body is a primary objective of human locomotion. This study aimed to explore UT kinematics according to gender and level of training and in relation to lower limb run patterns described through the presence of: overstriding, crossover, excessive protonation, and pelvic drop. Lower body variables chosen to describe running pattern were those that are frequently modified during gait-retraining with the goal of reducing injury risk. Eighty-seven recreational runners (28 females and 59 males, age 41 ± 10 years) performed a one minute run test on a treadmill at self-selected speed. UT kinematics was measured using an inertial measurement unit, while run features were assessed through an optoelectronic system and video analysis. Accelerations and root-mean-square on mediolateral and anteroposterior axes, normalized using the vertical component of the acceleration, were estimated to describe UT stability. Results showed no significant differences in the normalized UT acceleration root-mean-square according to gender and level of training as well as according to the presence of overstriding, crossover, and excessive protonation. The only running strategy studied in this work that showed a significant relationship with UT stability was the presence of excessive pelvic drop. The latter was significantly associated (*p*=0.020) to a decrease in the normalized acceleration root-mean-square along the mediolateral direction. Although the excessive pelvic drop seemed to have a positive effect in stabilizing the upper body, concerns remain on the effect of a poor control of the pelvis on the biomechanics of lower limbs. Results obtained confirm the hypothesis that the lower body is able to respond to varying impact load conditions to maintain UT stability.

## 1. Introduction

Running is one of the most popular recreational physical activities in the world, as it provides substantial health benefits at minimal expense [1]. Inertial measurement unit (IMU) is a sensor equipped with a triaxial accelerometer gyroscope and/or magnetometer, leading to a direct detection of the linear acceleration and angular velocity of the body segment to which they are attached. Accelerometers have been adopted in human joint kinematics studies since 1990s [2, 3] by attaching the sensors on foot, shank, thigh, and pelvis. Recent development and refinement in the technology have made IMUs less cumbersome, more economic, and ecological representing an alternative respect to the traditional 3D motion capture [4]. In recent years, the use of those sensors has been extended to the analysis of sport performances [5] and in particular of running gait [6]. Related research studies in the running field, employing an IMU system, focused mainly on lower limb kinematics [6] with several different purposes, such as describing the running pattern [7], investigating the epidemiology and risk factors for injuries [8], assessing the effect of biomechanical interventions on kinetic, kinematic, and spatiotemporal running variables during rehabilitation from running injuries [9], just to name a few. On the contrary, upper body biomechanics in runners has been poorly investigated, and reported measurements are mostly derived from triaxial accelerometers placed on the lower trunk, in the attempt to describe the center of mass kinematics [1, 10]. However, it has been recognized that the dynamic stability of the upper body is a primary objective of human locomotion. Low and smooth accelerations of the upper body are considered as characteristics of a stable gait [1] that could be linked to lower energy consumption and reduced risk of injury [1, 11]. Upper trunk stability during walking and running results from attenuation mechanisms of the oscillations caused by the lower limb movements [12] and influences the transmission to the head of the shock provoked by the ground reaction force (GRF) [13]. During running gait, the collision of foot with ground generates the resultant GRF necessary for forward propulsion and support against gravity [1]. GRF provokes a shock from the lower to the upper body, along the kinetic chain [1, 13, 14] that is dissipated by the combination of passive (e.g., deformation of ligaments, muscle oscillation, increase in knee flexion, and limited protonation of the foot) and active mechanisms (e.g., increased muscle activation). Acceleration at any anatomical location depends on the shock level of attenuation at all points distal and from intensity and direction of GRF [1]. The pelvis and the spinal column play an important role in determining shock absorption. This attenuation manifests itself in the fact that the resultant acceleration tends to decrease going from tibia, to the pelvis, to head level. Level and number of repetition of this shock on musculoskeletal structures contribute to modify chronic running injury risks [1]. Even if it is recognized as a role of lower body kinematics in modulated shock transmission to the upper body [1, 15, 16] a thorough investigation on this topic have not been undertaken in running gait yet. Kawabata et al. measured lower and upper trunk (UT) accelerations in different phases of the running gait cycle, but they did not take into consideration lower limb movements [17]. So far, only a few attempts have been done to describe upper body kinematics and its relationship with lower limb movements. Mercer et al. assessed the characteristics of shock attenuation during high-speed running, concluding that shock attenuation increases linearly with running speed, and changes in running kinematics are characterized primarily by changes in the stride length [14]. Specific lower limb running pattern has been observed to alter intensity of the initial shock and intensity and direction of the GRF, having a certain influence on the level of shock transmitted to the upper body and its stability [18–20]. For example, the initial shock provoked by foot-ground collision during running can be modified by initial contact foot-strike pattern (rearfoot or forefoot) [19]. Some authors observed an influence of crossover gait and level of foot protonation on the mediolateral component of the GRF [21–24]. Other factors that are supposed to influence trunk stability during gait are gender and level of training, but studies exist only with regard to lower trunk level or walking gait [11, 24]. However, to the best of the authors knowledge, no study directly investigated the relationship between acceleration of the UT and gender, level of training, and lower limb and pelvis run pattern, described through the foot-strike pattern, presence of overstriding, crossover, excessive protonation (EPR), and pelvic drop (EPD), which have already been demonstrated to influence intensities and direction of the GRF, shock transmission, and running-related injuries [7, 18–20, 25–28]. This study aims to fill this gap in the literature by characterizing UT kinematics by exploring differences in the UT kinematics according to gender and level of training and in relation to lower limb run pattern. The proposed analysis will be conducted by using an IMU placed on the UT.

## 2. Methods

### 2.1. Participants and Experimental Procedure

87 recreational runners (28 females and 59 males) volunteered to participate in the study. Participants signed a written informed consent. The study was approved by the Don Gnocchi ethic committee. Inclusion criteria were being engaged in a running program (at least two sessions a week with a minimum continuous running time of 20 minutes per session), being free from injuries in the last two months and being free from chronic musculoskeletal diseases.

Subjects were requested to run on a treadmill following a previously developed protocol [28]. Five minutes of warm-up and familiarization with the treadmill and one minute of run test (approximately 150 running cycles) were carried out by each subject. Participants were asked to run at a self-selected speed [28, 29]. Self-selected speed was chosen to control for differences in running kinematics that could arise at a speed different from the habitual training one (either too low or too high) [17]. All participants wore conventional, neutral running shoes to avoid potential influence shoes which may have on gait mechanics [29].

### 2.2. Instrumentation

**Figure 1:**
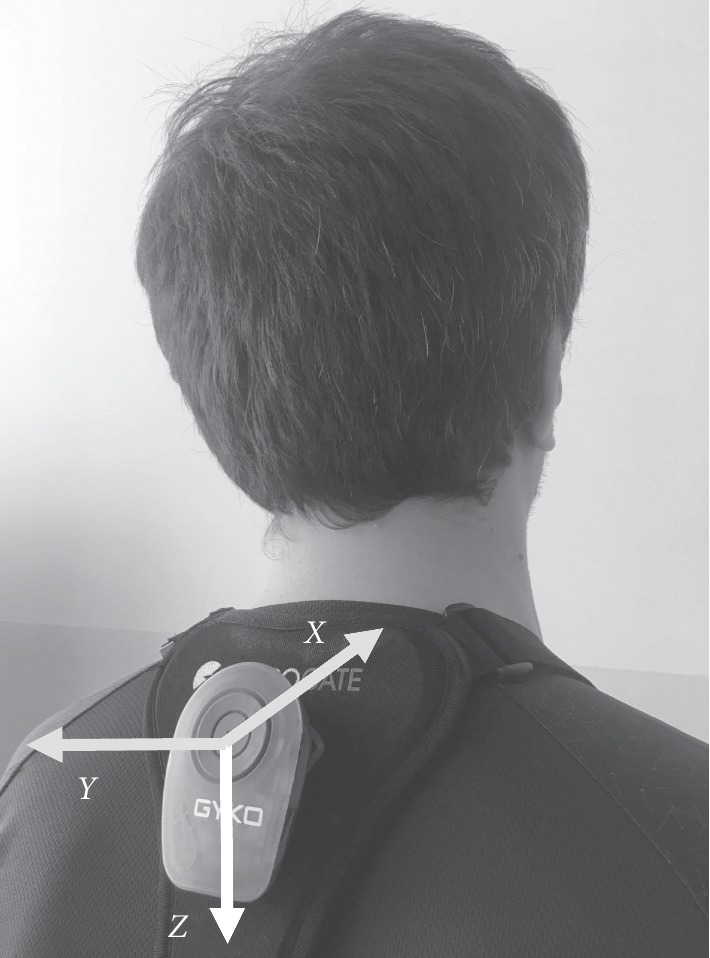
Placement of the inertial measurement unit at the upper-trunk level and axis orientation.

Running analysis was carried out by using a high-resolution IMU (Gyko, Microgate, Bolzano, Italy) in combination with a marker-less optical system (Optogait, Microgate, Bolzano, Italy) and video analysis. The IMU was equipped with a triaxial accelerometer, a triaxial gyroscope, and a triaxial magnetometer to measure linear acceleration, angular velocity, and magnetic field. The IMU was perpendicularly attached to an elastic harness provided with the system through automatic buttons. By using the manufacturer-provided harness, the IMU was positioned with the *y*-axis parallel to the back midline between the scapulae and the *z*-axis parallel to the line drawn between T1 to T5. This procedure was followed to approximately align the IMU to the anatomical axes, identified through anatomical landmarks and guided by the harness provided by the manufacturer. The sensor was oriented with the *X*-axis pointing backwards, representing the anterior-posterior direction (AP), the *Y*-axis pointing to the left representing the mediolateral direction (ML) and the *Z*-axis pointing downwards representing the vertical direction (*V*) (Figure 1). Data were sampled at a frequency of 1000 Hz.

The Optogait system is made up of two couples of transmitting and receiving bars. Each bar contains 96 LEDs that transmit on an infrared frequency with the same number of LEDs on the opposite bar. The system detects interruptions in the transmission between the bars. The first interruption of the LED signal during contact time is defined as “Initial Contact” and the portion of contact time during which the foot interrupts the maximum number of LEDs is defined as “Midstance phase.” Bars were placed on the sides of the treadmill tape, at the ground level, and fixed with adhesive tape to avoid movements caused by treadmill vibrations. The IMU and the Optogait systems were synchronized automatically by software provided by the manufacturer. In addition, the Optogait system was synchronized with two high-resolution cameras to film the frontal and sagittal planes of the participants. The videos were used to make frame-by-frame running video analysis provided by Kinovea software (version 0.8.15). Relevant landmarks for the videographic reference were marked using colored tape. Markers were placed on the low back at the level of the 5th lumbar (L5) vertebra and bilaterally by the external malleolus, the midline of the distal heel counter, the head of the fibula, the lateral condyle of the femur, the great trochanter, and the posterior iliac superior spine.

### 2.3. Data Analysis and Measured Parameters

Data processing was performed using custom procedures written in MATLAB R2017a (MATLAB, Mathworks Inc., Natick, MA, USA). The IMU reference frame was first rotated to have the *Z*-axis aligned with the gravity vector during the static postures at the beginning of each trial [30]. The acquired acceleration signal was lowpass-filtered using a 2nd order zero-lag Butterworth filter. The cutoff frequency value was set to 20 Hz, after having checked the frequency content of signals collected. The acceleration root-mean-square (aRMS) was then calculated along each axis to quantify the trunk kinematics.

RMS of the acceleration was computed for each separated axis using the following equation:

(1) $$\begin{array}{c} \text{aRMS}=\frac{\sqrt{x_{1}^{2}+x_{2}^{2}+\ldots +x_{N}^{2}}}{N}, \end{array}$$

where *x* is the acceleration measured along the AP, *V*, or ML axis and *N* is the number of samples. In this analysis, aRMS was computed over the entire duration of the signal.

The RMS calculated along the *V*-axis was then used to normalize the RMS contribution along AP and ML axes by using the following formula:

(2) $$\begin{array}{c} \text{naRMS}\_i=\frac{\text{aRMSi}}{\text{RMS}\_V}, \end{array}$$

where *i* is the respective axis: AP or ML. This calculation resulted in a unitless ratio for each axis and was performed to account for influence of running speed on trunk acceleration [31].

Lower body variables chosen to describe running pattern were those that are frequently modified during gait-retraining with the goal of reducing injury risk [27] and that can be easily identified through a frame-by-frame video analysis, namely, the presence of rearfoot strike, overstriding, crossover, EPR, and EPD. Rearfoot strike, overstriding, and crossover were assessed to describe the running pattern at initial contact, EPR, and EPD at midstance. Running gait phases were identified by the use of the Optogait system, and the presence or not of the selected running patterns was detected by video analysis with manual digitation. Criteria used to assess the presence of rearfoot strike, overstriding, crossover, EPR, and EPD by 2D video analysis referred to those used in literature [7, 18, 27, 28]. In particular, rearfoot refers to initial contact made by the heel in which the heel lands before the ball of the foot, a midfoot strike refers to an initial contact in which the heel and the ball of the foot land quasisimultaneously, while in a forefoot strike the ball of the foot lands before the heel [7]. Overstriding occurs when, at initial contact, the knee is completely extended, and the ipsilateral foot lands anteriorly to the pelvis [27]. Markers placed on the external malleolus, the head of the fibula, and the lateral condyle of the femur and the great trochanter were used to observe the complete extension of the knee and the foot placement compared with the pelvis (assumed to be on the same axis of the great trochanter). Crossover occurs when both feet land on the contralateral side of the body midline during a gait cycle [18]. The body midline was identified with the vertical line passing through the marker placed on the L5 vertebra. EPR and EPD describe running strategy during the midstance phase [27]. EPR is a triplanar motion of the subtalar joint characterized by a flattening of the medial arch and a hypermobile midfoot [27]. EPR was assessed by evaluating, through 2D video analysis, heel eversion, as reported by Souza et al. [27]. Presence of EPD was ascertained by evaluating the position of the iliac crest on the stance limb, which in EPD, is characterized by an excessive elevation relative to the contralateral iliac crest during the first half of the stance phase [27].

### 2.4. Statistical Analysis

Statistical analysis was performed using the STATA 10.0 Software, from Stata Corporation (College Station, Texas, USA). Data were firstly tested for normality using the Shapiro–Wilk test and then analyzed accordingly. Since acceleration measured at the lower trunk level was observed to be influenced by the training status [11], with more trained subjects showing lower aRMS values, differences in normalized aRMS were investigated between subjects with different training levels. The criterion to be considered more trained runners was running more than 50 kilometers per week [10]. An independent *t*-test, with a *p* value of 0.05 as significance level, was used to compare the two groups. The magnitude of the difference was assessed through the effect size (*d*) which was interpreted as low, if <0.3, medium, if comprised between 0.3 and 0.5, or large, if >0.5. The same analysis was conducted to investigate differences in normalized aRMS between males and females and subjects with different running patterns.

Then, normalized aRMS on each axis was entered, as the dependent variable, into a multiple linear regression model in which variables previously shown to be associated with aRMS with a *p* value <0.05, was considered as independent variables, while age, sex, weight, and height were assumed as confounding factors.

## 3. Results

Participants' characteristics are given in Table 1. Runners were aged between 19 and 61 years with a larger number of males (59/87, 68%). More than half of the participants exhibited a rearfoot strike pattern (67%), a crossover behavior (54%), and an EPR (57%), whereas most of them were free from overstriding (94%) and from EPD (64%). Because of the low number of subjects presenting overstriding, the parameter was excluded from further analysis. Normalized aRMS values observed along the AP and ML axes were comparable.

Table 2 shows normalized aRMS values measured along each axis in the male and female groups. Mean normalized aRMS for males was 0.203 ± 0.051, 0.212 ± 0.033, respectively, along the AP and ML directions. Similar values were observed in females (AP: 0.194 ± 0.048 and ML: 0.207 ± 0.024, *p* > 0.05; detailed *p* values are reported in Table 2).

More trained runners ran on average 62 km per week. When normalized aRMS was assessed in this group, values obtained were 0.196 ± 0.045 along the AP direction and 0.216 ± 0.026 along the ML direction. Averaged kilometer ran per week was 31 for the less-trained participants. Trunk accelerations measured in this group were not significantly different (*p* > 0.05, detailed *p* values are given in Table 2) from those measured in the most-trained group, with a normalized aRMS value of 0.202 ± 0.053 and 0.208 ± 0.032 along the AP and ML axes, respectively. Since no significant differences in the normalized aRMS were observed between males and females and between more- and less-trained runners, data were pooled in further analyses. A significant difference was found in normalized aRMS measured along the ML direction between runners who exhibited a tendency towards crossover and those who did not (*p*=0.023, *d* = 0.5, Table 3), with a higher acceleration value measured in the second group.

Along the same axis, acceleration of the UT was significantly lower in runners who had EPD compared with those that did not (*p*=0.032, *d* = 0.5). Even when sociodemographic variables (age, sex, weight, and height) were included in the analysis as confounding factors, the presence of EPD remained significantly associated to a decrease in the aRMS along the ML direction (*p*=0.020, Table 4).

## 4. Discussion

The aims of this study were to assess whether, in recreational runners, UT loading response and stance might be affected by sex, level of training, and lower body running pattern. The aim was accomplished by using an IMU, and a quantitative assessment of UT kinematics was obtained.

In our sample, no differences were found in terms of the aRMS on the AP and ML planes between men and women. The result is in agreement with findings observed for walking gait in a study of Mazzà et al., aiming to investigate gender differences in gait patterns [24]. Mazzà et al., by examining shock attenuation during gait, found similar values for the UT aRMS in men and women, when the gait was performed both at comfortable and fast speed.

In our group of runners, the level of training did not affect the measured aRMS (Table 2). Different from what observed in this study, Mc Gregor et al. reported significant differences in lower trunk acceleration when comparing a group of more-trained runners (semiprofessional athletes) with a group of recreational runners [11]. Discrepancies observed in the two studies may in part be the result of the different locations of the sensor (upper vs. lower trunk) and the consequent different shock attenuations provided by the intervertebral disks. However, more likely, the cause of different results obtained lays in the higher gap in training present between the two groups studied by Mc Gregor compared with the groups investigated in this study, with the latter including only recreational runners [11]. Indeed, more trained athletes develop a greater capacity to stabilize the trunk segments during running; however, this difference seems to become significant only in presence of an advanced level of training (professional and semiprofessional athletes).

Runners involved in this study did not show differences in UT aRMS, neither in the AP nor in the ML plane, in presence of different lower limb strategies with regard to the contact phase. The only running strategy that showed a significant relationship with UT stability was the EPD. To the best of the authors' knowledge, there are no studies in literature that have investigated possible correlations between running patterns and UT accelerations; thus, a direct comparison of results across studies is not possible.

Concerning different strike patterns, Gruber et al. investigated a group of habitual rearfoot runners and a group of habitual forefoot runners with the purpose of determining differences in the head and tibial acceleration signal power and shock attenuation [19]. What arose was that rearfoot strikers had significantly higher peak accelerations at the tibia level in comparison with forefoot strikers, while accelerations at the head level were not different between the two groups. Authors concluded that the body has the capacity to manage a range of impulsive loads derived by the shock generated from the GRF, in order to protect the head from excessive acceleration and to guarantee the stability of the oculovestibular system, which seems to be in accordance with results obtained in this study [19, 32]. In fact, none of the lower limb running patterns analyzed in the present study did not influence UT accelerations, even if literature shows that they influence direction and/or intensity of the resultant GRF.

This principle might apply also to EPR, and this might be the reason why a relationship between EPR and UT stability was not detected in our runners. A certain degree of protonation is physiological, and it contributes to loading absorption [33]. However, literature is not in agreement on the effect of physiological and excessive protonation on the ML component of the GRF.

The only running strategy studied in this work that showed a significant relationship with UT stability was the EPD. Running patterns presenting EPD seemed to be associated to lower ML aRMS at the UT level during the entire gait cycle. Previous investigations confirmed this finding by observing an association between excessive pelvic drop and the reduction of shoulder and head displacements during the weight acceptance phase of running [20, 34, 35]. It is conceivable that pelvis and spinal column play a determinant role in managing and absorbing the shock derived from GRF, even if lower limb running pattern influences its direction and intensity. Our study supports the hypothesis of Lim at al. that hip can be used by runners to meet upper body stability demands [13]. Moreover, Mazzà et al., in a study on walking gait, observed that the spinal column at all levels plays an important role in attenuating the shock provoked by the GRF, transmitted from lower limbs to the head, and this attenuation is already effective at the shoulder level (Mazzà). The same mechanism it is probably working for running pattern, and further studies are needed to corroborate this hypothesis.

Although the EPD seems to have a positive effect in stabilizing the upper body, concerns remain on the effect of a poor control of the pelvis on the biomechanics of lower limbs. As pointed out by Powers et al., in activities characterized by a single foot contact phase, such as running, the presence of EPD might cause the GRF vector passing lateral with respect to the knee joint center [34]. The valgus moment that originates in this case places a tensile strain on the medial soft tissue restraints of the knee, which represents one of the districts more prone to injuries in recreational runners [34, 36].

Future studies should investigate whether the possible protective role of pelvis drop on UT and oculovestibular system stability, has a negative effect on lower limb kinematics and might, eventually, lead to a higher risk of injuries.

The authors acknowledge that the use of a treadmill represents one of the limitations of this study. The main reason for testing athletes on a treadmill was the need to evaluate the protonation and pelvic drop parameters which otherwise would have been difficult to detect through a video analysis performed overground. An additional limitation is represented by the absence of a functional calibration and the manual alignment of the IMU with the anatomical axes. Although having followed the manufacturer's instructions and having performed a tilt correction of the acceleration signal through the gravity vector, a perfect alignment with the anatomical axes can not be guaranteed.

Moreover, kinematic differences between overground and treadmill running were deemed as acceptable for the purposes of this study, according to data reported in literature [14, 30].

Further investigations conducted in an ecological setting would surely add an important contribution to the results obtained in this study.

## 5. Conclusions

In our sample of recreational runners, UT stability did not appear to be affected by the gender, the level of training, and lower limb strategies during contact phase. However, it was found to be related to the compensation mechanisms of the pelvis on the ML plane. Results obtained confirm the hypothesis that the lower body is able to respond to varying impact load conditions to maintain UT stability.

## Figures and Tables

**Table 1:** Sample's characteristics.

Subjects' characteristics	Mean ± SD
Sex (%*F*)	31%
Age (yrs)	41 ± 10
Height (cm)	174 ± 8
Mass (kg)	69 ± 10
Running velocity (m/s)	10.6 ± 1.5

Initial contact characteristics	
Rearfoot (%*Y*)	67%
Overstriding (%*Y*)	6%
Crossover (%*Y*)	54%
Midstance characteristics	
Excessive pronation (%*Y*)	57%
Excessive pelvic drop (%*Y*)	36%
Upper trunk acceleration patterns	
Anteroposterior aRMS (g)	0.278 ± 0.067
Mediolateral aRMS (g)	0.294 ± 0.045
Vertical aRMS (g)	1.400 ± 0.094
Anteroposterior naRMS^*∗*^	0.191 ± 0.046
Mediolateral naRMS^*∗*^	0.201 ± 0.027

^*∗*^Normalized acceleration root-mean-square.

**Table 2:** Normalized acceleration root-mean-square (naRMS) values measured along each axis in the male and female group and in the more trained and less trained ones.

		Males	Females	*p*	More trained	Less trained	*p*
		*n* = 59	*n* = 27		*n* = 30	*n* = 57	
	v (m/s)	3.1 ± 0.4	2.7 ± 0.3	**<0.001**	3.1 ± 0.5	2.9 ± 0.4	**0.004**
	km/sett	41.1 ± 19.2	41.9 ± 15.8	**0.856**	61.7 ± 11.4	30.7 ± 9.6	**<0.001**

UT acceleration variables							
Anteroposterior naRMS^*∗*^		0.203 ± 0.051	0.194 ± 0.048	0.420	0.196 ± 0.045	0.202 ± 0.053	0.606
Mediolateral naRMS^*∗*^		0.212 ± 0.033	0.207 ± 0.024	0.461	0.216 ± 0.026	0.208 ± 0.032	0.231

^*∗*^Normalized acceleration root-mean-square.

**Table 3:** Normalized acceleration root-mean-square (naRMS) values of lower limbs parameters measured along each axis.

	Rearfoot		*p*	Crossover		*p*	Excessive pronation		*p*	Excessive pelvic drop		*p*
UT acceleration variables	Yes	No		Yes	No		Yes	No		Yes	No	

	*n* = 58	*n* = 29		*n* = 43	*n* = 37		*n* = 45	*n* = 34		*n* = 29	*n* = 52	
Anteroposterior naRMS^*∗*^ median (interquartile range)	0.211 (0.07)	0.186 (0.08)	0.115	0.186 (0.08)	0.216 (0.07)	0.053	0.196 (0.07)	0.210 (0.08)	0.759	0.181 (0.09)	0.206 (0.06)	0.344

Mediolateral naRMS^*∗*^ median (interquartile range)	0.214 (0.04)	0.210 (0.02)	0.089	0.202 (0.02)	0.217 (0.04)	**0.023**	0.213 (0.04)	0.203 (0.05)	0.109	0.202 (0.03)	0.214 (0.04)	**0.032**

^*∗*^Normalized acceleration root-mean-square.

**Table 4:** Multiple linear regression describing the relationship between excessive pelvic drop and crossover with mediolateral naRMS, adjusted for age, sex, weight, and height.

Final model: obs = 86; prob > chi^2^ < 0.036; *R*-square 0.170			
	Unstandardized coefficients	Standard error	*p*
naRMS^*∗*^ mediolateral			
Constant	0.301	0.114	0.010
Sex	−0.013	0.010	0.217
Age	0.000	0.000	0.517
Height	−0.001	0.001	0.473
Weight	0.000	0.001	0.798
Crossover	−0.011	0.006	0.103
Excessive pelvic drop	−0.016	0.007	**0.020**

^*∗*^Normalized acceleration root-mean-square.

## Data Availability

The data used to support the findings of this study are available from the corresponding author upon request.
